# Participant Perceptions of Continued Mental Health First Aid (MHFA) Engagement, Help-Seeking Behavior, and Stigma Reduction: A Mixed-Methods Pilot Study

**DOI:** 10.3390/healthcare14142064

**Published:** 2026-07-09

**Authors:** Michael A. D. Smith, Jennifer Marazzo, Lillian D. Williams, Eric Fishon, Josh Muhammad, LaTika Muhammad, Venecia Williams, Ashlee Shands, Arleen Downer-Reid

**Affiliations:** 1 European Business School of Barcelona (ENEB), C/Llull 321–329, 08019 Barcelona, Spain; dr.michaeladsmith@gmail.com; 2 Academy of Certified Brain Injury Specialists, Brain Injury Association of America, Fairfax, VA 22031-1931, USA; jmarazzo228@gmail.com; 3 American College of Healthcare Executives, Chicago, IL 60606-6698, USA; lillianwilliams924@gmail.com; 4 Department of Science in Stress and Health Management, Hellenic American University, Nashua, NH 03063, USA; efishon@hauniv.edu; 5 Department of Biochemistry & Molecular Biology, University of Florida, Gainesville, FL 32611, USA; 6 Department of Nursing, The University of Alabama, Tuscaloosa, AL 35487, USA; latika.muhammad@gmail.com; 7 Phoenix Health Research Institute, Inc., Albuquerque, NM 87123, USA; vnelson3077@hotmail.com (V.W.); lowery.ashlee@gmail.com (A.S.); arldreid@gmail.com (A.D.-R.)

**Keywords:** Mental Health First Aid (MHFA), stigma reduction, help-seeking behavior, attitudinal change, mixed-methods study, pilot study, community support

## Abstract

**Highlights:**

**What are the main findings?**

- Participants reported continued confidence, help-seeking engagement, and application of MHFA skills following training.
- The self-stigma scale demonstrated strong internal consistency, while qualitative measures showed increased self-awareness, stigma reduction, increased advocacy, professional empowerment, and perceived institutional validation of mental health priorities.

**What are the implications of the main findings?**

- MHFA training may support community-based mental health literacy and help-seeking engagement among participants.
- Integrating Mental Health First Aid into organizational wellness and training initiatives may promote longer-term cultural and behavioral change.

**Abstract:**

Background/Objectives: Mental Health First Aid (MHFA) is an internationally recognized training program designed to improve mental health literacy, reduce stigma, and train non-clinicians with supportive skills to assist individuals experiencing psychological distress. Although prior research has demonstrated trainees’ short-term improvements in knowledge and attitudes, less is known about their post-certification perceptions. Here, we assess the likelihood of trainees’ continued application of MHFA concepts several months after certification. The current study examined the participants’ self-reported perceptions of the behavioral and attitudinal outcomes three to six months following training, including, help-seeking behaviors, self-efficacy, stigma-related attitudes, and functional application of intervention skills. Methods: A mixed-methods, cross-sectional design was employed using a structured web-based survey administered to MHFA-trained participants. The survey included demographic items, Likert-scale measures of confidence, behavioral engagement, and stigma-related attitudes, as well as open-ended qualitative prompts. Quantitative analyses included descriptive statistics, reliability testing using Cronbach’s alpha, exploratory factor analysis, assumption diagnostics, and nonparametric hypothesis testing using Mann–Whitney U tests. Qualitative responses were analyzed using thematic analysis to identify recurring patterns related to long-term training impact. Results: Participants reported increases in self-confidence and behavioral engagement following MHFA training. More than 80% of participants indicated they had recommended professional mental health services to others after certification. Psychometric evaluation demonstrated strong internal consistency for the six-item self-stigma scale (α = 0.90), with a unidimensional factor structure explaining 60.4% of the variance, whereas the public stigma scale showed weaker internal reliability (α = 0.45). Qualitative themes included increased self-awareness, stigma reduction, advocacy behavior, professional empowerment, and institutional validation of mental health priorities. Conclusions: The findings suggest participants perceived continued confidence, reduced internalized stigma, and engagement in supportive mental health behaviors following MHFA training. These findings should be interpreted cautiously given the cross-sectional design, self-reported measures, and highly engaged sample.

## 1. Introduction

Mental health remains a significant global public health concern, with many individuals experiencing mental health challenges across their lifetime. Despite increasing awareness, stigma and limited mental health literacy continue to act as major barriers to help-seeking behavior. Individuals often avoid seeking professional support due to fear of judgment, internalized stigma, and insufficient knowledge of available treatment options [1].

Mental Health First Aid (MHFA) has emerged as a widely implemented training program designed to improve mental health literacy, reduce stigma, and equip non-clinicians with the skills necessary to support individuals experiencing psychological distress. Originally developed in Australia, MHFA has since been adopted internationally and aims to enhance recognition of mental health conditions, promote early intervention, and encourage appropriate help-seeking behaviors [2,3,4,5,6,7,8].

Previous research has demonstrated that MHFA training is linked to improved knowledge, increased self-confidence, and more supportive attitudes toward individuals with mental health conditions. In addition, MHFA has been linked to increased willingness to seek professional counseling and to recommend help-seeking to others [3,8,9]. The program’s structured approach, including the ALGEE action plan, provides participants with practical tools to respond effectively to mental health concerns.

Beyond immediate outcomes, MHFA may also be connected to broader psychosocial factors, including stigma reduction and longer-term behavioral change. Social and self-directed stigma remain critical barriers to accessing mental healthcare. By improving understanding and promoting empathetic engagement, MHFA has the potential to reduce stigmatizing attitudes and normalize mental health conversations [10,11,12].

In particular, there is a need to better understand how MHFA influences sustained behavioral engagement, self-efficacy, and attitudes over time. The aim of this pilot study was to examine participant perceptions regarding the relationship between MHFA training, stigma-related attitudes, help-seeking behaviors, and mental health-related activities following certification. Additionally, higher levels of self-efficacy were expected to be associated with increased engagement in MHFA-related behaviors, consistent with social cognitive theory [1,2].

This study examined the reported longer-term impact of MHFA training on help-seeking behavior, self-efficacy, and stigma-related attitudes using a mixed-methods pilot design. Although the study utilized a cross-sectional design and retrospective self-report measures rather than longitudinal follow-up, the findings may provide insight into participants’ self-reported sustained application of MHFA-related behaviors over time.

The aim of this pilot study is to examine how MHFA training influences mental healthcare, reduces stigma, and relates to changes in mental health-related activities among participants following MHFA training. The main aim is to evaluate the participants’ positive changes in knowledge, attitudes, and self-reported behavior correlated with MHFA training. As a secondary goal, the study seeks to explore whether the reported changes may diminish over time. Additionally, the study assesses the relationship between demographic characteristics, time since training, and self-efficacy concerning the sustainability of MHFA practices in real-life conditions. Mental health literacy is one field that has gained increasing importance, and MHFA is a globally implemented program aimed at empowering laypersons to identify, support, and refer individuals experiencing mental health crises [5,6]. This investigation contributes to ongoing efforts to evaluate the continued applications of MHFA beyond immediate post-training improvements.

## 2. Materials and Methods

### 2.1. Study Design

A mixed-methods, delayed post-survey, cross-sectional design was employed to examine the reported post-training outcomes of Mental Health First Aid (MHFA) training on help-seeking behavior, self-efficacy, and stigma-related attitudes 3–6 months after training. The inclusion of self-efficacy as a study outcome was informed by Bandura’s theory of self-efficacy, which posits that individuals with greater confidence in their abilities are more likely to engage in supportive helping behaviors [13]. The study utilized a structured web-based survey incorporating both quantitative and qualitative components to allow for data triangulation and a more comprehensive understanding of participant experiences [14].

### 2.2. Study Population and Sampling

Participants were recruited from a national online community of certified MHFA practitioners through Facebook advertising, email invitations via MHFA alumni networks, and outreach to mental health advocacy groups. The final analytic sample consisted of 70 participants (*n* = 70) who met the inclusion criteria and completed the survey. The sample consisted primarily of certified MHFA instructors, reflecting a highly engaged subset of MHFA trained individuals.

Although the sample was non-random and based on voluntary participation, multiple recruitment strategies were used to enhance diversity across geographic regions and professional backgrounds. Recruitment efforts included posting study participation announcements in publicly accessible MHFA-related social media groups and distributing survey invitations through publicly available instructor contact information listed on the MHFA website.

### 2.3. Inclusion and Exclusion Criteria

Participants were eligible if they (1) were at least 18 years of age, (2) were English or Spanish speakers, and (3) had completed MHFA training through the National Council for Mental Wellbeing in the United States. There was no restriction placed on time duration between certification and assessment of perceived long-term effects. Participants were excluded if surveys were incomplete or if training was completed outside the United States to ensure consistency in training standards.

### 2.4. Ethical Considerations

Informed consent was obtained electronically prior to participation. The consent form outlined the study purpose, voluntary nature of participation, and assurances of anonymity. No personally identifiable information was collected. The study received Institutional Review Board (IRB) approval and was conducted in accordance with the ethical principles outlined in the Belmont Report and applicable federal regulations governing human subjects’ research [15].

### 2.5. Data Collection Procedures

Data were collected utilizing a web-based survey administered over a 12-week period (15 October 2025, to 16 February 2026). The survey was accessible via mobile and desktop devices and required approximately 10–15 min to complete. No financial compensation was provided. Reminder emails were sent periodically to maximize response rates. Recruitment and survey materials were provided in both English and Spanish to enhance accessibility.

### 2.6. Survey Instrument and Measures

The survey instrument was developed collaboratively by the research team for this study using Qualtrics and reviewed for content validity by three certified MHFA instructors and one mental health researcher. The instrument included multiple-choice, Likert-scale, and dichotomous (yes/no) items across five domains:

(1) Demographics and background;
(2) Recall of MHFA training content;
(3) Perceived self-efficacy;
(4) Behavioral engagement;
(5) Attitudes toward stigma and help-seeking.

Internal consistency was assessed using Cronbach’s alpha [16]. The six-item self-stigma scale demonstrated strong reliability (α = 0.90), whereas the public stigma scale showed lower reliability (α = 0.45).

### 2.7. Statistical and Qualitative Analysis

Quantitative data were analyzed using IBM SPSS Statistics, Version 29 (IBM Corp., Armonk, NY, USA). Descriptive statistics (means, standard deviations, frequencies, and percentages) were calculated for all variables. Internal consistency was assessed using Cronbach’s alpha. Exploratory factor analysis (EFA) using principal axis factoring was conducted to examine the dimensional structure of stigma measures. Sampling adequacy was assessed using the Kaiser–Meyer–Olkin (KMO) measure and Bartlett’s test of sphericity.

Assumption testing included the Shapiro–Wilk test for normality and Levene’s test for homogeneity of variance. Due to violations of normality, nonparametric tests were applied. Mann–Whitney U tests were used to examine group differences in stigma, confidence, and help-seeking behaviors. Missing data was minimal and handled using pairwise deletion.

### 2.8. Qualitative Analysis

**Figure 1:**
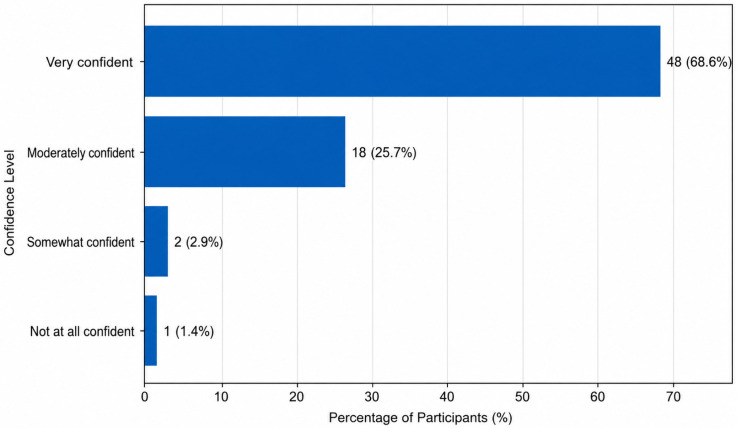
Self-reported confidence after MHFA training. Note. Figure displays the distribution of participants’ confidence level percentages in identifying and addressing mental health symptoms following MHFA training (*N* = 70). Most participants reported moderate or high levels of self-efficacy in identifying and addressing mental health concerns following certification.

Qualitative responses were analyzed using a six-phase thematic analysis approach as described by Braun and Clarke [17]. Data were coded inductively, followed by the development of higher-order themes to capture recurring patterns related to stigma, behavioral change, and participant experiences following MHFA training. The distribution of participants’ self-reported confidence after MHFA training is presented in Figure 1.

**Figure 2:**
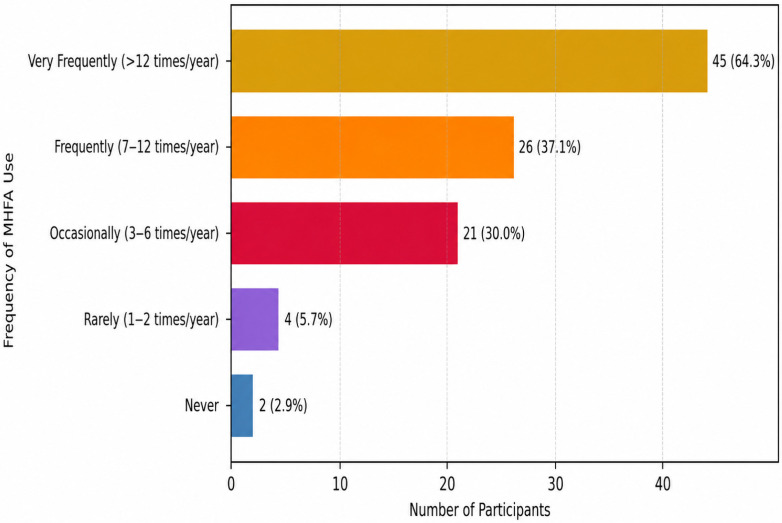
Frequency of using MHFA techniques in real-life scenarios. Note. Figure shows the percentage/frequency with which participants reported applying MHFA techniques in real-life situations following certification (*N* = 70). Most participants indicated using MHFA strategies frequently or very frequently in professional or personal contexts.

Participants’ reported frequency of applying MHFA techniques following certification is shown in Figure 2.

**Figure 3:**
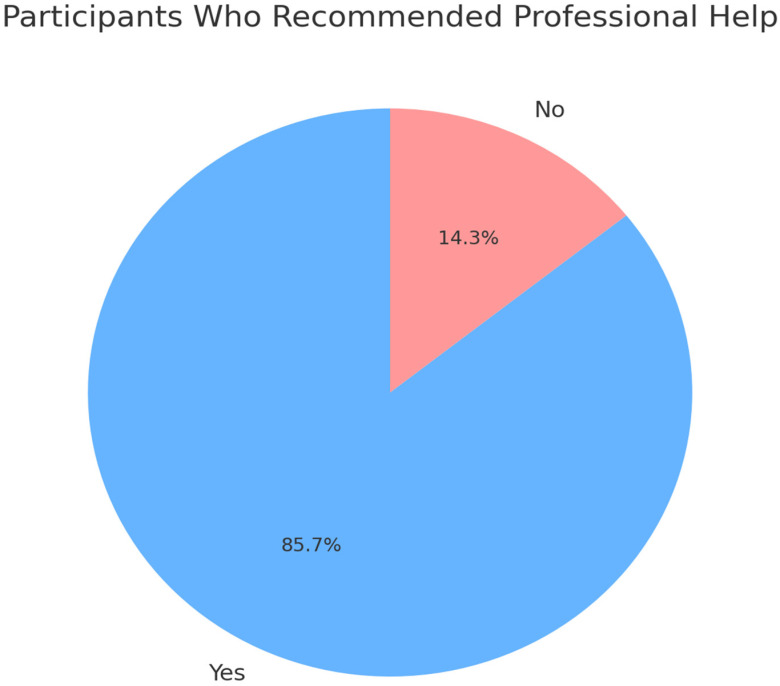
Participants who recommended professional help. Note. Figure presents the proportion of participants who reported recommending professional mental health support to others after completing MHFA training (*N* = 70). The majority of respondents indicated that they had encouraged someone to seek professional help.

The proportion of participants who reported recommending professional mental health support to others after completing MHFA training is presented in Figure 3.

### 2.9. Data Collection and Thematic Analysis Process

Qualitative Data Collection

The qualitative data analyzed in this study were collected through open-ended questions embedded in a delayed post-survey, administered approximately three to six months after participants completed MHFA training. The survey aimed to evaluate longer-term perceptions of MHFA’s impact, particularly regarding stigma reduction, help-seeking behaviors, and personal or professional application. Participants provided responses voluntarily, and no incentives were offered.

Thematic Analysis Framework

A six-phase thematic analysis, as described by Braun and Clarke [17], guided the interpretation of qualitative responses:

- Familiarization with the data—Reading and re-reading responses for immersion.
- Generating initial codes—Noting semantic patterns and assigning preliminary labels.
- Searching for themes—Organizing codes into overarching conceptual patterns.
- Reviewing themes—Refining and collapsing themes based on internal coherence and distinctiveness.
- Defining and naming themes—Clarifying the scope and meaning of each theme.
- Producing the report—Integrating findings into a coherent narrative with illustrative quotes.

### 2.10. Coding Strategy and Trustworthiness

Initial coding was performed manually using Excel spreadsheets, allowing inductive themes to emerge organically while also incorporating deductive codes informed by the research question. Codes were tagged and grouped iteratively. A short audit trail documenting analytic decisions and reflective notes (memoing) was maintained to ensure transparency and trustworthiness. Peer debriefing was conducted with an external reviewer to confirm theme accuracy and reduce bias. Any differences in interpretation that emerged during the review process were resolved through discussion and refinement of thematic definitions to improve consistency and analytic credibility. Although no formal inter-coder reliability metric was used, trustworthiness was enhanced through thick description, reflexivity, and verbatim participant quotes.

## 3. Results

Recruitment was facilitated through universally accessible social media announcements and survey invitations distributed publicly through MHFA-related contact information. Therefore, the total number of individuals exposed to the study invitation could not be readily determined. A formal survey response rate could not be calculated for the same reason. This limitation is common in open, web-based recruitment designs where the sampling frame is not fixed and the number of eligible individuals who viewed or received the invitation is unknown.

To address this limitation, the study reports the final analytic sample size rather than a response rate and interprets findings as exploratory and pilot in nature. The use of voluntary participation may have introduced self-selection bias, as individuals with a stronger interest in mental health, MHFA training, or stigma reduction may have been more likely to participate. Therefore, findings should not be generalized to all MHFA-certified individuals. Future studies should consider using a defined sampling frame, direct invitation tracking, unique survey links, and documented invitation counts to allow calculation of response rates and stronger assessment of nonresponse bias.

The professional profile of participants (*N* = 70) reflects a concentration within social and educational sectors. A higher percentage of participants were affiliated with social services (38.6%) and education (25.7%), while representation from the veteran/military sector was minimal (1.4%) (see Table 1). Participant experience levels indicate a predominantly experienced sample. Among respondents who reported certification tenure (*n* = 66), 80.3% had held certification for more than two years, whereas only 3.0% had less than three months of experience.

Instructional reach further highlights the advanced level of participants. Among those reporting teaching experience (*n* = 67), nearly half (47.8%) had trained more than 250 individuals, while only 3.0% had taught fewer than 10 students. Certification frequency (*n* = 64) suggests a pattern of continued engagement, with most participants reporting one (37.5%) or two (39.1%) certification cycles. Higher-frequency recertification (four or more times) was relatively uncommon (see Table 1).

### 3.1. Demographic Characteristics of Participants

The study sample consisted of 70 participants, with valid responses ranging from 62 to 63 across demographic variables due to missing data. Demographic characteristics, including gender, age, education, race/ethnicity, and selected cross-tabulations, are presented in Table 2. Most participants identified as female (87.3%), while 9.5% identified as male and 3.2% identified as non-binary or third gender. The largest age group represented was 55–64 years (27.0%).

In terms of educational attainment, most participants held a master’s degree (53.2%), followed by bachelor’s (21.0%) and doctoral degrees (16.1%). Most participants identified as Caucasian (66.1%), with African American (19.4%) and Hispanic (14.5%) participants representing smaller proportions of the sample. Cross-tabulation analyses demonstrated that female participants represented the majority across racial/ethnic categories, while educational attainment was distributed across multiple age groups (see Table 2).

### 3.2. Stigma Scale Reliability Analysis

Internal consistency analyses were conducted to evaluate the reliability of the public stigma scale. After removal of one item with zero variance, the remaining four-item scale demonstrated weak internal consistency (Cronbach’s α = 0.45), suggesting that findings related to public stigma should be interpreted cautiously within this exploratory pilot sample.

The six-item self-stigma scale demonstrated strong internal consistency (Cronbach’s α = 0.90), indicating excellent reliability across items and supporting the internal coherence of the self-stigma construct within this sample.

### 3.3. Preliminary and Assumption Testing

Before the testing of the hypothesis, the data underwent screening in terms of missing values, normality, and homogeneity of variance. Loss of data was insignificant among outcome variables, with a range of 5.7 percent (stigma scores) to 11.4 percent (help-seeking likelihood), and were overcome by pairwise deletion in non-parametric tests. The test of homogeneity of variance was done by the use of the Levene test. The homogeneity of variances on personal experience groups was observed in the case of stigma (*F*(1, 64) = 0.32, *p* = 0.57), as well as in the case of confidence (*F*(1, 62) = 0.14, *p* = 0.71) (see Table 3).

The Shapiro–Wilk tests were used to measure normality. Stigma scores did not differ significantly with normal distribution as indicated in Table 3 (*W* = 0.96, *p* = 0.051). Conversely, confidence scores were also in major violation of the normality assumption (W = 0.55, *p* < 0.001). As a result of this violation, all outcome variables were subjected to non-parametric tests to provide consistency of analysis.

**Correlation of Personal Experience and Stigma**

The comparison of differences between participants who had a personal experience of mental health difficulties and those who did not was performed through a Mann–Whitney U test. Table 4 presents the descriptive statistics by personal experience group. Participants with personal experience had a slightly higher stigma score (M = 2.93) than those without personal experience (M = 2.39). Table 5 summarizes the Mann–Whitney U test results, which indicated that the difference between groups was not statistically significant (U = 209.00, p = 0.21).

**Correlation of Personal Experience and Confidence**

Mann–Whitney U test was used to investigate the difference between the confidence in identifying and addressing mental issues based on the status of personal experience. It was found that there was no significant trend in favor of more confident participants who had a personal experience (*M* = 4.65) over those who did not experience it (*M* = 4.44) (U = 176.00, *p* = 0.08) (see Table 4 and Table 5).

**Correlation of Personal Experience to Likelihood of Help-Seeking**

The Mann–Whitney U test was used to test the relationship between personal experience and the probability of seeking professional help. According to Table 5, the difference between the participants who had personal experience (M = 4.45) and those who did not (*M* = 4.44) (*U* = 214.50, *p* = 0.57) was not significant. Table 4 demonstrates the group means.

**Correlation between Personal Experience and Frequency of Help-Seeking**

Lastly, a Mann–Whitney U test was used to compare help-seeking frequency after MHFA training. The findings showed that there was no statistically significant difference between the participants with and without personal experience (*M* = 1.64 and 1.56) (U = 241.50, *p* = 0.91) (see Table 4 and Table 5).

Participants represented a range of professional sectors, with the largest groups affiliated with social services (38.6%), education (25.7%), and healthcare (15.7%). Other workplace affiliations accounted for 18.6% of the sample, while veteran/military participants represented 1.4%. In terms of race and ethnicity, most participants identified as Caucasian (66.1%), followed by African American (19.4%) and Hispanic (14.5%).

**Figure 4:**
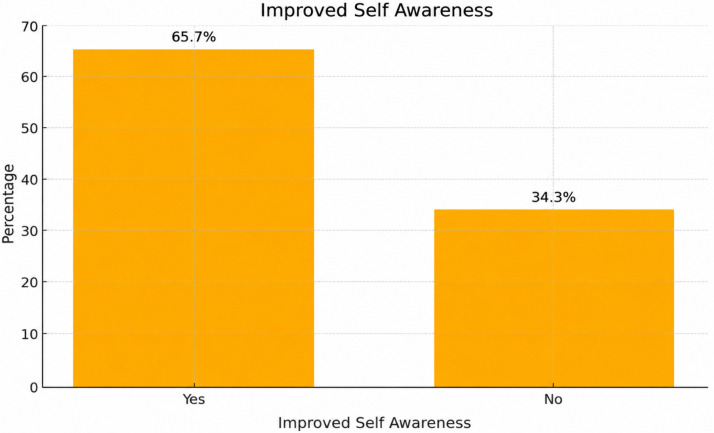
Self-reported increase in mental health self-awareness after MHFA training. Note. Figure displays participant responses regarding increased mental health self-awareness following MHFA training (*N* = 70). A substantial majority reported greater self-awareness after completing the certification.

**Figure 5:**
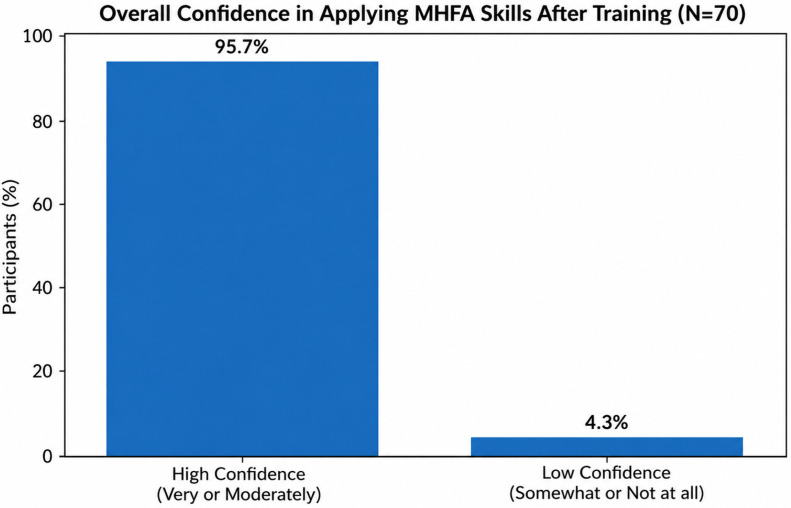
Overall confidence in applying MHFA skills. Note. Figure presents the overall distribution of participant confidence in applying MHFA skills following certification (*N* = 70). Responses were grouped into high confidence (very confident or moderately confident) and low confidence (somewhat confident or not confident). The majority of participants reported high confidence in applying MHFA techniques.

In terms of participant self-awareness and confidence in their own mental health, as well as their ability to identify and address mental health symptoms, a substantial majority of participants (85.7%) reported greater self-awareness following MHFA training. More than 90% rated themselves as either moderately confident or very confident in addressing mental health symptoms. The distribution of participants’ self-reported increase in mental health self-awareness following MHFA training is presented in Figure 4. Figure 5 presents the overall distribution of participant confidence in applying MHFA skills following MHFA certification.

**Figure 6:**
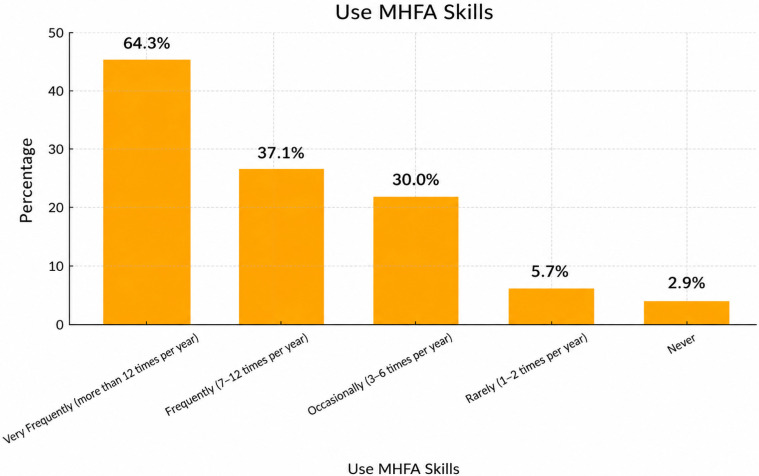
Frequency of real-life use of MHFA skills. Note. Figure displays the frequency with which participants reported applying MHFA techniques in real-world situations following certification (*N* = 70). Most respondents indicated using MHFA strategies either frequently or very frequently.

When asked about the real-life application of MHFA techniques, most participants reported using these skills frequently or very frequently in professional or personal contexts. The frequency with which participants reported applying MHFA skills in real-life situations following certification is presented in Figure 6.

### 3.4. Behavioral Impact Analysis

**Figure 7:**
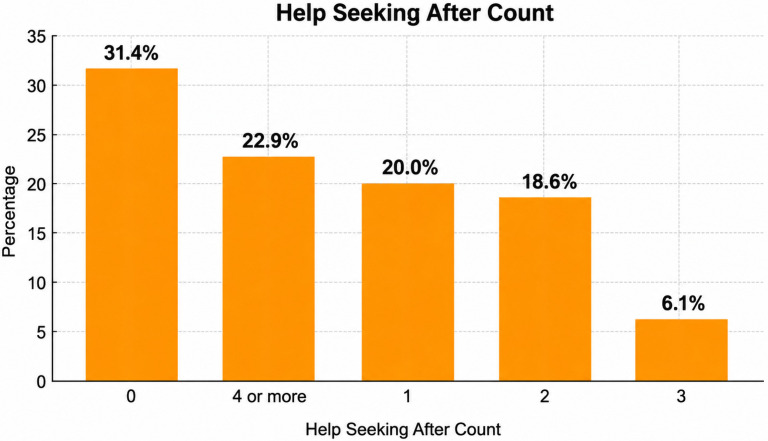
Frequency of help-seeking after MHFA training. Note. Figure displays the frequency with which participants reported seeking professional help for their own mental health challenges after completing MHFA training (*N* = 70). Responses ranged from none to four or more instances.

Participants reported varying frequencies of seeking professional help for their own mental health challenges after MHFA training. Responses ranged from no help-seeking after training to four or more instances, with a substantial proportion reporting one or more episodes of help-seeking. The frequency with which participants reported seeking professional mental health support for their own mental health challenges following MHFA training is presented in Figure 7.

After completing MHFA training, most participants (85.7%) reported recommending that someone seek professional mental health support. These findings indicate that MHFA-trained participants frequently encouraged help-seeking among others in their personal or professional communities.

### 3.5. Participant Demographics and Response Overview

#### 3.5.1. Total Participants and Qualitative Response Count

Out of the 70 participants, 57 submitted at least one qualitative response, resulting in 133 total responses across the open-ended survey questions. These responses varied in depth and detail, ranging from brief reflections to more extended narratives suitable for thematic analysis. 

#### 3.5.2. Response Depth and Patterns

The average length of open-text responses was approximately 40 to 70 words, with some entries exceeding 100 words. Richer and more personal narratives often came from individuals in education, counseling, or crisis intervention roles, suggesting deeper reflection and application of MHFA content in their daily work.

#### 3.5.3. Optional Comments Noted

Several participants voluntarily shared emotionally resonant anecdotes—such as referring someone to therapy for the first time, recognizing signs in themselves, or feeling validated in their professional roles. Representative quotes from participants are included in subsequent sections to illustrate the identified themes.

### 3.6. Qualitative Findings and Analysis

Thematic analysis of open-ended survey responses identified five themes describing participants’ perceptions of the influence of Mental Health First Aid (MHFA) training on attitudes, behaviors, and professional practices related to mental health. Representative participant quotations are included to illustrate each theme.

**Theme 1: Increased Self-Awareness and Awareness of Distress**

Participants frequently described an increased ability to recognize signs of psychological distress in themselves and others. Responses indicated greater awareness of emotional states and early indicators of mental health concerns.

One participant stated, “Training helped me know what anxiety is like in my students as well as in me.” Another participant noted that the training helped them recognize when they were experiencing stress and prompted greater self-monitoring.

**Theme 2: Reduction in Stigma and Shame**

Participants described changes in perceptions of mental health and reported greater openness in discussing mental health concerns. Several responses reflected reduced negative perceptions and increased empathy toward individuals experiencing mental health challenges. Eighteen participants specifically indicated that MHFA training influenced their personal or professional attitudes toward stigma.

One participant shared, “I used to think that depression was a weakness before MHFA. It is like any other disease that I am learning to understand now.” Another participant stated, “I no longer feel ashamed to speak about my mental health; now I ask other individuals how they feel about themselves.”

**Theme 3: Modeling and Advocacy Behavior**

Participants reported engaging in behaviors such as initiating conversations about mental health, providing emotional support, and discussing personal experiences.

One participant stated, “I’ve begun speaking freely about my therapy process to debunk it from being taboo. It’s incredible how many people opened up after.” Another participant reported, “Now I don’t just wait—I reach out when I see something’s off with someone.”

**Theme 4: Professional Empowerment**

Participants described increased confidence in responding to mental health concerns within professional settings. Responses frequently referenced greater comfort using skills and language gained during MHFA training, particularly among educators, counselors, and frontline professionals.

One participant explained, “Before MHFA, I would stay silent, worried I might make things worse. Now, I step in with confidence—I feel like I have the tools, and that makes all the difference.”

**Theme 5: Validation of Mental Health as a Priority**

Participants indicated that MHFA training reinforced the importance of mental health within their organizational environments. Responses reflected perceptions that mental health concerns were receiving increased attention and recognition.

One participant noted, “MHFA made me feel like the school finally cared about this issue.” Another participant stated, “For the first time, I felt like leadership recognized mental health as important.”

## 4. Discussion

### 4.1. Interpretation of Key Findings

The above five emergent themes, including increased self-awareness and institutional validation, describe how MHFA training may contribute to participants’ beliefs, behaviors, and professional identity. Instead of offering separate results, these themes form a symbiotic narrative of change: participants described how MHFA provided tools to respond to mental distress while also influencing their understanding of mental health and the way they talked about it. This is also in line with general behavioral science trends that seek to normalize language around mental health and reduce stigma [10]. The findings suggest the potential for MHFA to support greater discussion and awareness of mental health within workplace and community settings.

Although previous studies have consistently demonstrated MHFA’s short-term effects, fewer studies have explored participants’ perceptions of longer-term experiences following MHFA training [11,18]. The present findings contribute to the growing literature examining how MHFA may relate to mental health awareness, stigma reduction, and supportive intervention practices across professional and community settings. These findings suggest that participants attributed continued application of MHFA-related attitudes and behaviors following training. However, several quantitative comparisons did not demonstrate statistically significant group differences, and the findings should therefore be interpreted as exploratory participant perceptions rather than evidence of definitive behavioral effects.

Participant responses regarding modeling and positive self-confidence in dealing with crises conform with the central tenets of empowerment theory, which postulates that people develop a sense of control over the decisions and actions that affect their lives [19]. MHFA training may function as a skills-based intervention while also supporting participants’ view of increased agency and advocacy. Most assessments of MHFA have concentrated on short-term variations in knowledge or attitudes, but the current findings provide insight into participants’ perceptions of continued application of supportive behaviors several months following MHFA training. These findings are consistent with emerging longitudinal evidence, which suggests that MHFA may support perceptions of continued mental health engagement, communication patterns, and self-efficacy, which are key elements of sustainable mental health promotion [18].

This study contributes to the growing body of evidence exploring participant views of Mental Health First Aid (MHFA) training and its potential influence as a widely implemented intervention [20,21]. While much of the existing literature has focused on immediate post-training outcomes such as increased knowledge and reduced stigma, these findings suggest perceived continued behavioral and cultural influence of MHFA concepts several months following training. Participants’ reflections suggest ongoing engagement with MHFA-informed approaches. It is important to note that these views were reported by a highly engaged MHFA sample composed largely of instructors and helping professionals, which may have influenced the generally positive nature of participant responses.

The study findings should be interpreted within the context of several important sampling limitations. The participant pool was composed primarily of highly experienced Mental Health First Aid (MHFA) instructors, with 84.3% identifying as instructors and 80.3% reporting more than two years of MHFA experience. As a result, the sample reflects a particularly engaged subgroup of the MHFA community rather than the broader population of MHFA trainees or workplace participants. Individuals who choose to become instructors may possess stronger pre-existing commitments to mental health advocacy, greater confidence in applying MHFA skills, and more favorable perceptions of the program than typical trainees.

Additionally, participation in the survey was voluntary, creating the potential for self-selection bias whereby individuals with positive training experiences may have been more likely to respond. These factors may have contributed to more favorable assessments of MHFA’s long-term impact and may limit the transferability of the findings to less experienced trainees, general workforce populations, or individuals with minimal ongoing engagement in mental health initiatives. Consequently, the results should be viewed as evidence of MHFA’s perceived long-term influence among a highly involved and experienced cohort rather than as definitive evidence of outcomes across all MHFA participants. Future research employing larger and more diverse samples, including non-instructors and recent trainees, is needed to determine the extent to which these findings are generalizable across broader populations.

The integration of quantitative and qualitative findings strengthened the interpretation of the study results through methodological triangulation. Quantitative findings demonstrated high levels of self-reported confidence, frequent use of MHFA skills, and supportive help-seeking behaviors, while qualitative responses provided contextual insight into how participants view these experiences within personal, professional, and organizational settings. Together, these findings offer complementary perspectives regarding participants’ application of MHFA-related knowledge, attitudes, and behaviors following training.

All outcomes were measured through participant self-report. Consequently, responses may have been influenced by recall bias, social desirability bias, or participants’ preexisting commitment to mental health advocacy. Objective behavioral measures were not available and future studies should incorporate observational, organizational, or longitudinal outcome measures to strengthen inference.

### 4.2. Stigma Reduction and Mental Health Literacy

Perceived reductions in stigma emerged as one of the most frequently reported themes. Participants acknowledged prior misconceptions, such as viewing mental illness as a personal weakness, and described how MHFA training was perceived to contribute to a shift toward more informed, compassionate, and biopsychosocial understandings of mental health. These findings are consistent with prior research demonstrating MHFA’s effectiveness in reducing stigma and promoting empathy [10,22]. Notably, many participants reported both attitudinal changes and engagement in supportive behaviors, with over 80% of participants reporting that they had supported or assisted someone experiencing mental distress. This aligns with previous studies indicating increased confidence and helping behaviors following MHFA training [4,11].

The enhancement of mental health literacy is consistent with the findings of Morgan et al. [23], who reported that MHFA training significantly improves mental health knowledge, reduces stigma, and increases helping behaviors. The thoughts of the participants also correlate with the evidence provided by Morgan, according to which the enhanced awareness and recognition of mental health symptoms were proven as the most significant benefits of MHFA interventions [24]. Cognitive changes of this nature are essential towards early intervention and self-care, especially in cases where there is extreme stress such as school-based or community health-based organizations.

Findings related to public stigma should be interpreted cautiously, as the public stigma scale demonstrated weak internal consistency within the current sample (α = 0.45), although qualitative responses consistently reflected themes of reduced stigma and increased openness toward mental health discussions. The findings are consistent with regard to Corrigan et al. [10], who have stated that psychoeducation interventions such as MHFA have an impact on reducing stigma in the community and by an individual based on reframing concepts of mental illness in terms of bio-psychosocial issues. Likewise, post-MHFA stigma reduction was especially conspicuous among individuals who had low original exposure to mental health education, which accords with the findings of Hadlaczky et al. [11]. Reducing internalized shame and external judgment, MHFA assists in normalizing help-seeking and humanizing psychological struggles.

These are behaviors that conform to the social learning theory proposed by Bandura, indicating learning through observation, modeling, and reinforcement [12]. The willingness of participants to express openness and support may reflect greater comfort engaging in mental health discussions within their professional environments. This phenomenon is correlated with the results of Hadlaczky et al. [11], who found that MHFA-trained individuals often serve as peer influences who help propagate discussions about mental health. These are particularly positive forms of advocacy in settings where stigma or silence has traditionally existed.

According to this institutional validation, the study carried out by LaMontagne et al. [25] paid considerable attention to the effect of organizational commitment in fostering employee well-being. Prior research suggests that organizational leadership may play an important role in supporting employee well-being and mental health initiatives. In turn, a recent study conducted by Geierstanger et al. concluded that the most effective outcomes of MHFA can be achieved when its implementation is addressed within the context of overall organizational wellness [26].

### 4.3. Advocacy, Self-Efficacy, and Organizational Culture

Participants also reported engaging in advocacy-related behaviors, including initiating conversations about mental health, modeling supportive attitudes, and encouraging others to seek professional help. These behaviors reflect increased self-efficacy and are consistent with social cognitive theory, which emphasizes the role of perceived capability in driving behavior [12]. Similarly, these findings align with empowerment theory, which highlights individual agency and perceived control as key drivers of sustained behavioral change [19].

Several participants described broader shifts within their organizational environments, including greater openness, reduced judgment, and a stronger sense of shared responsibility for mental health. Participants’ accounts suggested increased openness and supportive mental health communication within their organizational environments following MHFA training. MHFA has also been adapted across culturally diverse populations, highlighting the importance of context-specific implementation strategies [27,28]. This is supported by prior research identifying MHFA-trained individuals as informal peer leaders who promote mental health awareness and supportive practices [11,23,29].

Additionally, participants highlighted the value of the group-based training model, noting that shared learning experiences fostered trust, reduced stigma, and strengthened interpersonal connections. These findings support the notion that MHFA functions not only as an educational intervention but also as a team-building and culture-enhancing mechanism [26].

Despite these contributions, several limitations must be acknowledged. The reliance on self-reported data introduces the potential for recall and social desirability biases. Future research would benefit from triangulating self-report data with observational or longitudinal measures. Additionally, although participants represented diverse backgrounds, the sample may be biased toward individuals already interested in mental health topics, potentially limiting generalizability.

### 4.4. Practical Implications

Nevertheless, the findings have important practical implications. MHFA represents a low-cost, high-impact intervention that can support organizations in addressing mental health stigma, burnout, and employee well-being. Integrating MHFA into organizational wellness strategies may foster more supportive environments and facilitate earlier intervention [30]. Furthermore, these findings highlight the importance of longitudinal research to better understand the durability of MHFA-related outcomes and the potential need for refresher or booster training over time.

### 4.5. Limitations and Reflexivity

This study has several limitations. First, the reliance on self-reported data may introduce recall bias and social desirability bias. Second, the use of online recruitment methods, including Facebook and digital outreach, may limit generalizability due to potential sample homogeneity and the exclusion of individuals with limited internet access or those not engaged in online communities. Because recruitment occurred through publicly accessible social media postings and distributed survey invitations, the total number of individuals who viewed or received the study invitation could not be determined; therefore, a formal response rate could not be calculated.

Additionally, the study sample consisted largely of experienced MHFA instructors and professionals working in social services, education, and related helping professions. These participants may have already possessed more favorable attitudes toward mental health, help-seeking, and stigma reduction prior to participating in the study. As a result, the findings may reflect a response bias toward participants who were already highly engaged in mental health advocacy or supportive professional roles, thereby limiting broader generalizability to less experienced or non-helping populations.

The cross-sectional design precludes causal inference, and the directionality of observed relationships cannot be determined. Future longitudinal studies are recommended to examine sustained behavioral changes over time [31]. There are additional limitations relevant to measurement reliability and construct validity. The relatively small pilot sample limits the stability and interpretability of the exploratory factor analysis [EFA] findings, particularly for the public stigma scale, which demonstrated weak internal consistency. The weak reliability observed for the public stigma scale may reflect limited item consistency, reduced variability within the highly MHFA-engaged sample, or challenges tied to measuring public stigma perceptions using a brief exploratory instrument. Therefore, findings related to public stigma should be interpreted cautiously.

While the inclusion of qualitative data provides valuable contextual insight, these findings are not intended to be generalizable and may reflect the perspectives of more engaged participants. Reflexivity is also important in interpreting these findings, as researchers must consider how their own training, perspectives, and potential advocacy roles may have influenced data interpretation and reporting. The quantitative analyses were primarily exploratory and descriptive in nature, and the relatively small pilot sample limited the ability to conduct more advanced inferential modeling or subgroup analyses.

## 5. Conclusions

This study contributes to the growing evidence that Mental Health First Aid (MHFA) may serve as more than a short-term educational intervention. The findings suggest that participants reported continued mental health self-awareness, confidence in responding to psychological distress, reduced internalized stigma, and engagement in supportive help-seeking behaviors across diverse professional and community settings. Importantly, participants described changes in individual knowledge and attitudes as well as greater openness to discussing mental health within professional and community settings.

One of the most notable observations from this study was participants’ reports of greater openness toward mental health conversations. Participants also described greater confidence in responding to individuals experiencing distress. Participants frequently described increased willingness to intervene, recommend professional support, and openly discuss mental health concerns within their organizations and communities. These findings suggest that MHFA may strengthen both individual-level mental health literacy and collective organizational responsiveness.

The integration of quantitative and qualitative findings provided complementary perspectives on participants’ experiences following MHFA training. Quantitative results demonstrated high levels of self-reported confidence, frequent application of MHFA skills, and strong internal consistency within measures of self-stigma reduction. Qualitative themes provided contextual depth by illustrating how participants experienced increased professional empowerment, advocacy behaviors, and institutional validation of mental health as a priority. Together, these findings suggest that participants continued application of MHFA-related attitudes and behaviors beyond immediate post-training experiences.

At a broader public health level, the study emphasizes the necessity of scalable, community-based mental health interventions that support help-seeking practices. As mental health challenges continue to affect workplaces, schools, healthcare systems, and communities, MHFA may represent a practical and accessible strategy for strengthening mental health support networks outside traditional clinical environments.

Although the findings should be interpreted cautiously due to the study’s pilot design, voluntary sampling, self-reported measures, and the highly engaged nature of the sample, they nevertheless provide valuable insight into participants’ perceptions of the continued application of MHFA-related knowledge, attitudes, and supportive behaviors following training. Future longitudinal and multi-site studies will further evaluate the durability of these outcomes across more diverse populations and organizational contexts.

Ultimately, participant responses suggest that MHFA may extend beyond crisis-response training by encouraging greater openness, empathy, and engagement with mental health concerns in organizational and community settings.

## Figures and Tables

**Table 1:** Professional profile of study participants (*N* = 70).

Variable	Category	N	%
Instructor Status (*n* = 70)	Yes	59	84.3
	No	11	15.7
Workplace Affiliation (*n* = 70)	Social Services	27	38.6
	Education	18	25.7
	Healthcare	11	15.7
	Veteran/Military	1	1.4
	Other	13	18.6
Certification Frequency (*n* = 64)	Once	24	37.5
	Twice	25	39.1
	3 Times	10	15.6
	4 Times	2	3.1
	More than 5 Times	3	4.7
Certification Tenure (*n* = 66)	1 Month–3 Months	2	3.0
	3 Months–6 Months	3	4.6
	1 Year–18 Months	1	1.5
	18 Months–2 Years	7	10.6
	Over 2 Years	53	80.3
Instructional Reach (*n* = 67)	None	6	9.0
	1–10 Students	2	3.0
	26–50 Students	5	7.5
	51–100 Students	11	16.4
	101–250 Students	11	16.4
	More than 250 Students	32	47.8

**Table 2:** Descriptive statistics of participant demographics (*N* = 70).

Characteristic	Valid n	Missing n	Missing %	Most Frequent Category	Frequency	Percentage
Gender	63	7	10.0	Female	55	87.3
Age	63	7	10.0	55–64 years	17	27.0
Education	62	8	11.4	Master’s degree	33	53.2
Race/ethnicity	62	8	11.4	Caucasian	41	66.1
**Gender**		**African American**		**Caucasian**		**Hispanic**
Female		11		34		9
Male		1		5		0
Non-binary/third gender		0		2		0
**Age Category**		**Some College**	**Associate’s Degree**	**Bachelor’s Degree**	**Master’s Degree**	**Doctoral Degree**
25–34 years		0	1	2	4	0
35–44 years		1	1	3	9	2
45–54 years		1	0	1	8	3
55–64 years		1	1	6	7	2
65+ years		0	0	1	5	3

Note: Valid response counts varied across variables because of missing data. Percentages are based on valid responses for each demographic category. Cross-tabulation data are presented descriptively to summarize distributions of gender by race/ethnicity and age by education level within the sample. Due to small subgroup sizes, cross-tabulation findings should be interpreted cautiously and were not used for inferential statistical analysis.

**Table 3:** Assumption testing for outcome variables.

Outcome Variable	Test	Statistic	*p*
Stigma score	Shapiro–Wilk	*W* = 0.96	0.051
Confidence	Shapiro–Wilk	*W* = 0.55	<0.001
Stigma score	Levene’s test	*F*(1, 64) = 0.32	0.57
Confidence	Levene’s test	*F*(1, 62) = 0.14	0.71

**Table 4:** Descriptive statistics by personal experience group.

Outcome	No Experience (*n* = 11)	Personal Experience (*n* = 59)
Stigma score	2.39	2.93
Confidence	4.44	4.65
Help-seeking likelihood	4.44	4.45
Help-seeking frequency	1.56	1.64

**Table 5:** Mann–Whitney U tests comparing outcomes by personal experience.

Outcome	*U*	*p*
Stigma score	209.00	0.21
Confidence	176.00	0.08
Help-seeking likelihood	214.50	0.57
Help-seeking frequency	241.50	0.91

## Data Availability

The data presented in this study are available on request from the corresponding author. The data are not publicly available due to privacy and ethical restrictions related to research on human subjects.
